# Edrophonium Challenge Test for Blepharospasm

**DOI:** 10.3389/fnins.2016.00226

**Published:** 2016-06-06

**Authors:** Shinichi Matsumoto, Nagahisa Murakami, Hidetaka Koizumi, Masatoshi Takahashi, Yuishin Izumi, Ryuji Kaji

**Affiliations:** ^1^Department of Neurology, Shinko HospitalKobe, Japan; ^2^Department of Clinical Neuroscience, Institute of Health Biosciences, The University of Tokushima Graduate SchoolTokushima, Japan

**Keywords:** blepharospasm, dystonia, edrophonium

## Abstract

**Background:** Blepharospasm is typically diagnosed by excluding any secondary diseases and neuropsychiatric disorders, as specific tests for blepharospasm are currently unavailable. Since anticholinergic agents are used to improve the symptoms of dystonia, we hypothesized that edrophonium chloride, an acetylcholinesterase inhibitor, may make the symptoms of dystonia more apparent. Therefore, we examined whether an edrophonium challenge test would be useful for diagnosing blepharospasm.

**Methods:** We studied 10 patients with blepharospasm and 10 with hemifacial spasms (as disease controls). We administered edrophonium and saline in this double-blind study. Before and after the injection, we recorded the clinical signs using a video camera to assess the objective symptoms every 2 min. Ten minutes after the isotonic sodium chloride and edrophonium injections, the patients evaluated their subjective signs using a visual analog scale (VAS). The objective signs on the video recordings were scored by specialists who were blind to the treatment.

**Results:** The subjective and objective signs of the patients with blepharospasm were amplified by edrophonium. In contrast, the signs in patients with hemifacial spasms were not changed by the edrophonium challenge test.

**Conclusions:** The edrophonium challenge test may be used to diagnose blepharospasm. The study was registered with a ICMJE recognized registry, the UMIN-CTR, with the number UMIN000022557.

## Introduction

Dystonia is a clinical syndrome characterized by sustained muscle contractions that cause twisting and repetitive movements or abnormal postures (Fahn et al., 1998). Blepharospasm, which presents with abnormal eyelid closure, is caused by dystonia of the eyelids. In the clinic, blepharospasm is typically diagnosed by excluding any secondary diseases and neuropsychiatric disorders because of the unavailability of specific tests for blepharospasm. Usually, differential diagnosis can be performed easily by observing the symptoms, but sometimes the differences are difficult to identify because of the complicated symptoms of blepharospasm (Roberts et al., 2002; Hara et al., 2007). For example, in a previously encountered case, we administered an edrophonium injection to a patient with abnormal eyelid closure and diplopia, which resulted in improvement of the diplopia, but amplification of the abnormal eyelid closure. Complications of myasthenia gravis (MG) and blepharospasm were suspected in that case. Eyelid closure dysfunction in MG is related to abnormal functioning of the levator palpebrae superioris muscle due to deficient cholinergic transmission at the neuromuscular junction, while in blepharospasm, eyelid closure dysfunction is due to the strong involuntary contraction of the orbicularis oculi muscle.

In MG, the administration of edrophonium induces acetylcholine release, which then improves the symptoms. Thus, the edrophonium challenge test has been established as a diagnostic tool for MG. However, involuntary contraction of the orbicularis oculi muscle occurs due to blepharospasm or hemifacial spasm among other disorders. Although the clinical symptoms of hemifacial spasms resemble the symptoms of blepharospasm, the causes differ. Therefore, identifying the underlying causes of the spasms can help clinicians make an accurate diagnosis.

Edrophonium chloride is an acetylcholinesterase inhibitor with a rapid onset (approximately 30 s) and short duration (approximately 5 min). Suppression of dysfunctional eyelid closure by edrophonium is used to diagnose MG (Pascuzzi, 2003). Edrophonium is hydrophilic, with a limited capacity to cross the blood–brain barrier. In addition, edrophonium is rapidly excreted by the kidneys, suggesting limited direct efficacy in the central nervous system (Back and Calvey, 1972; Calvey et al., 1976; Gemmill et al., 1988). However, in our case, blepharospasm was amplified after administering the edrophonium injection, which suggests that edrophonium may be used to diagnose blepharospasm.

For the medical treatment of dystonia, Balash and Giladi concluded that high doses of trihexyphenidyl (anticholinergic agent) are effective, especially in the treatment of segmental and generalized dystonia in young patients (Balash and Giladi, 2004). Because it has been reported that anticholinergic agents improve the symptoms of dystonia, if the dysfunctional eyelid closure is caused by blepharospasm, then the symptoms may be made more apparent by edrophonium.

In this study, we examined whether an edrophonium challenge test would be useful for diagnosing blepharospasm. To accomplish this, we examined the clinical symptoms of 10 cases of blepharospasm after administering edrophonium injections. We also administered physiological saline as a control and examined cases with hemifacial spasms as a disease control.

## Methods

### Participants

The study included 10 patients with blepharospasm (2 men and 8 women; age range, 34–77 years; mean [standard deviation] age, 65.1 [11.9] years) and 10 patients with hemifacial spasms (4 men and 6 women; age range, 46–78 years; mean [standard deviation] age, 65.3 [9.1] years), who constituted the control group (Table 1). All participants were examined by a single movement disorder specialist (Shinichi Matsumoto), who performed general physical and neurological examinations, laboratory tests, and brain magnetic resonance imaging in order to exclude other causes of dystonia, including birth injury and head trauma. The patients with a history of heart disease and arrhythmia were excluded from participating in the study for their safety. All subjects underwent a 12-lead electrocardiogram examination and a chest X-ray before the study to exclude the presence of cardiac disease. This investigation was approved by the institutional ethics committee of Shinko Hospital. Written informed consent was obtained from all participants.

**Table 1:** **Clinical characteristics of patients with blepharospasm and hemifacial spasms**.

	**No. of subjects (female)**	Age (*SD*)^*^	**Age of onset (*SD*)**
Blepharospasm	10 (8)	65.1 (11.9)	57.0 (13.0)
Hemifacial spasms	10 (6)	65.3 (9.1)	59.0 (11.5)

* *Standard deviation*.

### Administration of edrophonium and evaluation of subjective symptoms

This was a double-blind study, as both the participants and evaluators were blind to the identity of the drug (saline or edrophonium). The protocol used for the edrophonium challenge test is shown in the Table 2. First, we intravenously injected isotonic sodium chloride (10 mL). Before and after the injection, we recorded the clinical symptoms with a video camera to assess the objective signs every 2 min (each video was 1 min long). Ten minutes after isotonic saline injection, the participants evaluated their subjective signs on a visual analog scale (VAS) (Mantha et al., 1993). We explained to the participants that the “left end” of the scale indicated the worst possible state, while the “right end” indicated a healthy state without blepharospasm or hemifacial spasm. Next, we administered edrophonium (10 mg in 9 mL of isotonic saline) and recorded the clinical signs as described above. Patients were directed to self-evaluate their signs separately from any side effects (Supplementary Videos 1, 2).

**Table 2:** **The protocol for administering the edrophonium challenge test in patients with movement disorders**.

**EVALUATION OF PATIENT SYMPTOMS**
1. Videotape (1 min) recording of objective symptoms before injection
2. Normal saline injection
3.2 min after injection: videotape recording
4.4 min after injection: videotape recording
5.6 min after injection: videotape recording
6.8 min after injection: videotape recording
7.10 min after injection: videotape recording
8. Visual analog scale (VAS) test (evaluation after normal saline injection)
9. Edrophonium injection
10.2 min after injection: videotape recording
11.4 min after injection: videotape recording
12.6 min after injection: videotape recording
13.8 min after injection: videotape recording
14.10 min after injection: videotape recording
15. VAS test (evaluation after edrophonium injection)
**EVALUATION OF THE VIDEO BY THREE MOVEMENT DISORDER SPECIALISTS**
1. We chose videos of the isotonic sodium chloride solution and edrophonium injections, randomly referred to as A or B
2. We chose the video acquired at 8 min after administration as A or B Because the symptoms changed the most around 8 min after edrophonium administration
3. The before injection video is shown
4. The after A injection video is shown
5. Modified VAS (the value of after A injection with before injection as midline)
6. The before injection video is shown
7. The after B injection video is shown
8. Modified VAS (the value of after B injection with before injection as midline) We evaluated “before and after A or B injection” as a set, as described above We randomly presented “before and after A or B injection” videos of blepharospasm and hemifacial spasm

### Evaluation of objective symptoms

Three movement disorder specialists (Nagahisa Murakami, Hidetaka Koizumi, and Masatoshi Takahashi), who were blind to the treatment and the purpose of the examination, independently evaluated the objective symptoms from the videos after the administration of the isotonic sodium chloride solution and edrophonium, which were randomly named A and B. We found that the symptoms changed the most around 8 min after edrophonium administration. For this reason, we chose to evaluate the 1-min videos that were acquired at 8 min after administration. We presented videos of “before and after injection of A or B” as a set. We randomly presented the “before and after injection of A or B” videos for blepharospasm and hemifacial spasm.

We used a modified VAS (mVAS) to evaluate the pre-administration baseline, which was set at 50 mm (midline); amelioration and exacerbation of symptoms were indicated by lower and higher numbers on the mVAS, respectively. The Jankovic score is often used to evaluate blepharospasm (Jankovic et al., 1982) and consists of 5 stages for both the frequency score and the severity score. However, clinically, the difference between the frequency score and the severity score was not clear, thus it really only evaluated five levels instead of 10. Because the Jankovic score was not suitable for evaluating the fine changes in blepharospasm after the edrophonium injection, we used the mVAS for the evaluations. In addition, because hemifacial spasms cannot be evaluated using the Jankovic score, we required another evaluation method.

The final control and drug mVAS scores for each patient were the sum of each specialist's score. The VAS and mVAS scores were measured from the right side, with a higher score indicating symptoms that were more severe.

### Statistical analyses

Mean VAS and mVAS scores were compared using the Wilcoxon rank sum test for paired continuous variables. We calculated the relative value as follows: VAS score after edrophonium injection—VAS score after saline injection. The relative values were also compared using the Wilcoxon rank sum test for paired continuous variables. All statistical tests were two-sided, and *P*-values less than 0.05 were considered statistically significant. Statistical analyses were performed using the SPSS statistical software (version 11.0 for Windows; IBM Corporation, Armonk, NY, USA).

## Results

**Figure 1:**
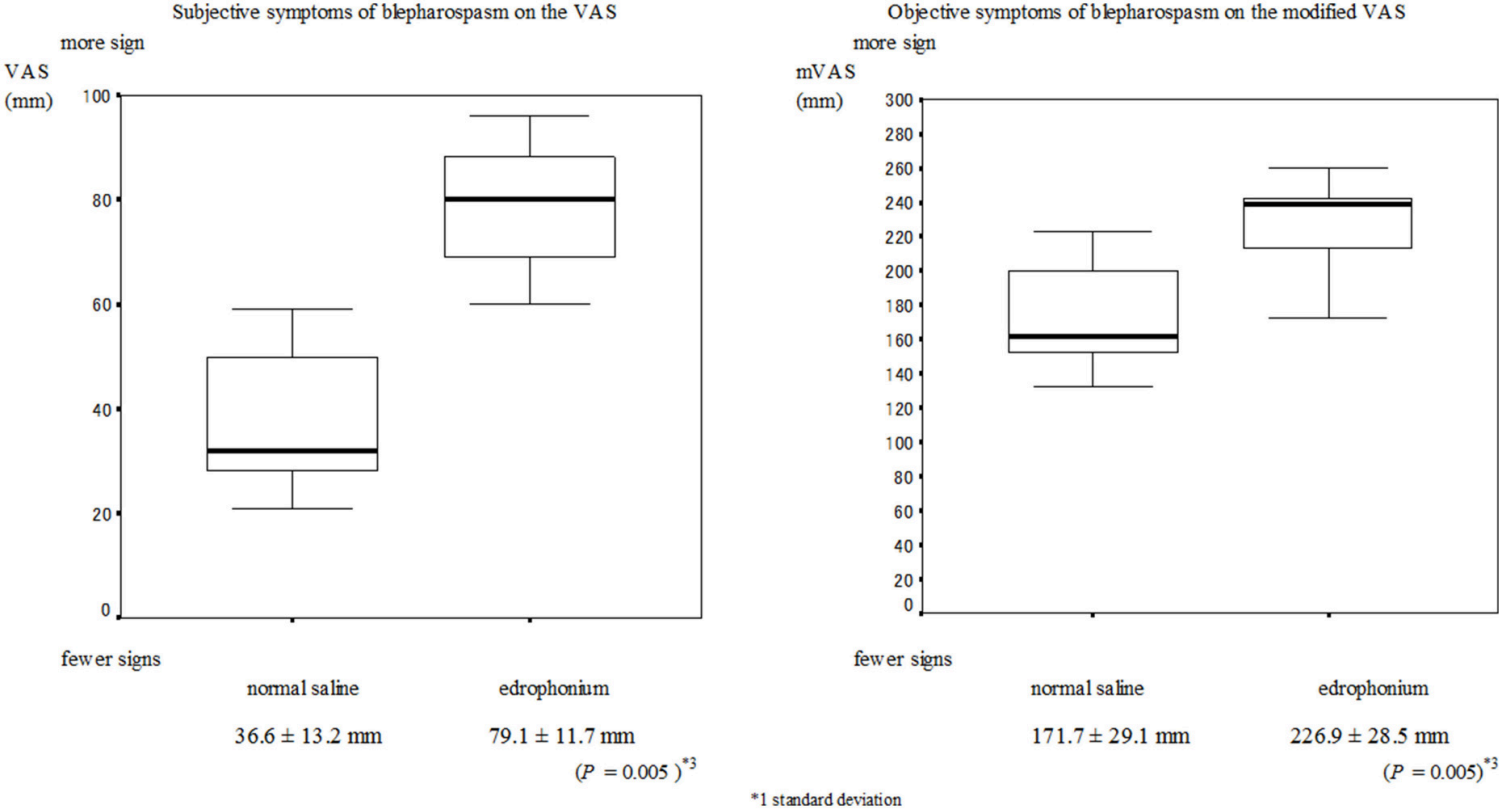
Mean and SD^*^1 values of the VAS^*^2 for the placebo injection session and the edrophonium injection session in patients with blepharospasm. **(A)** The VAS scores for the subjective symptoms after edrophonium injection were significantly higher than the scores after placebo injection (*P* = 0.005). **(B)** The modified VAS scores of the objective symptoms after edrophonium injection were significantly higher than the scores after placebo injection (*P* = 0.005).

**Figure 2:**
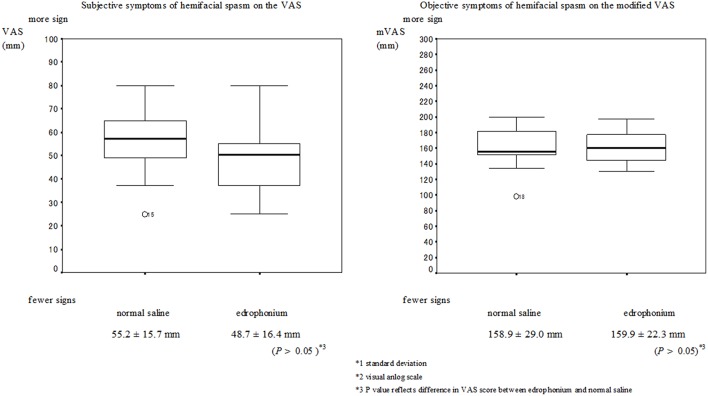
Mean and SD^*^1 values of the VAS^*^2 for the placebo injection session and the edrophonium injection session in patients with hemifacial spasm. There were no statistically significant differences in the VAS scores and modified VAS scores of patients with hemifacial spasms between the edrophonium and placebo injection groups.

**Figure 3:**
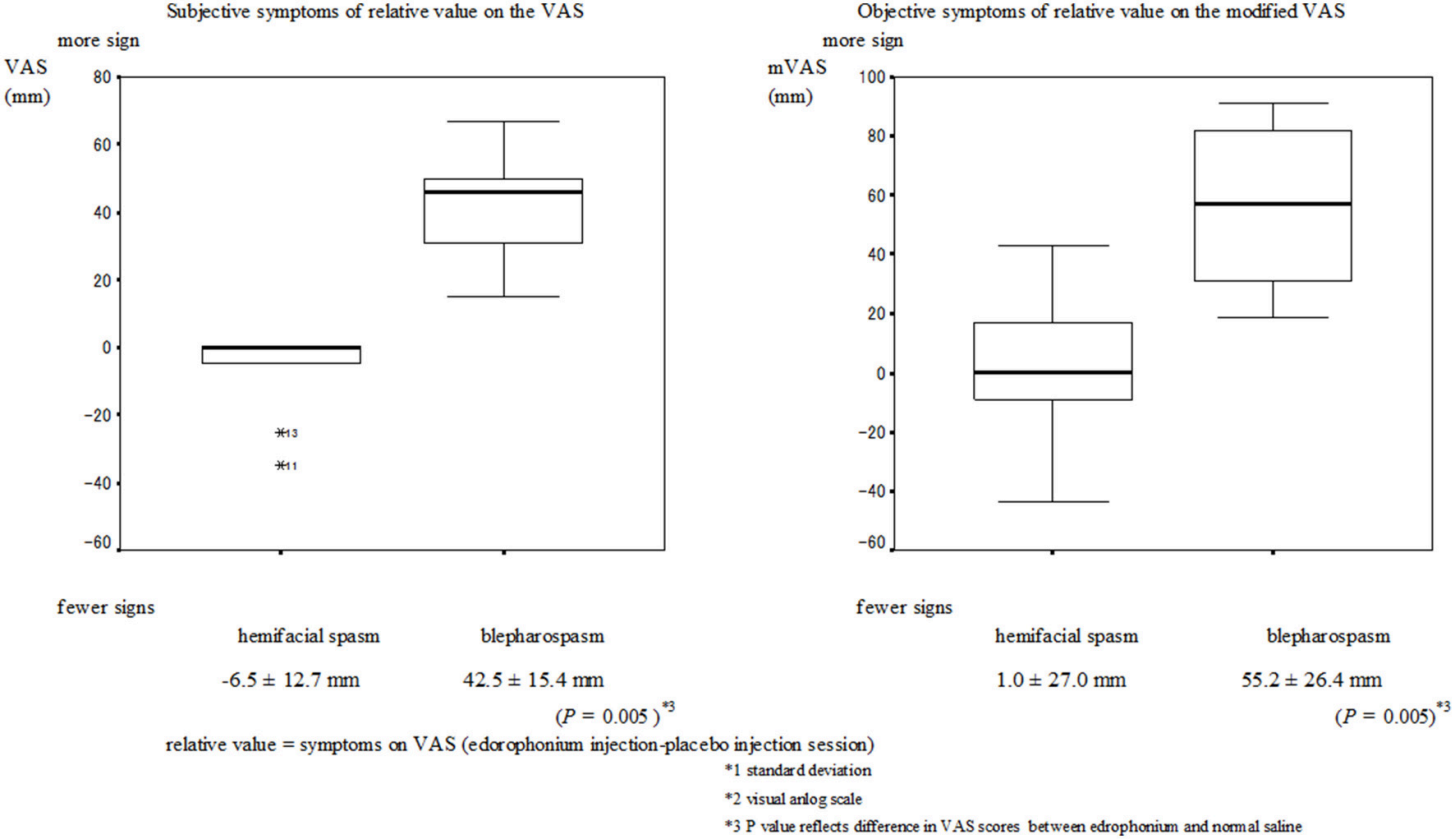
Mean and SD^*^1 of the relative values for the hemifacial spasm and blepharospasm. The VAS scores for the relative value of the subjective and objective symptoms in patients with blepharospasm were significantly higher than the scores in patients with hemifacial spasm (*P* = 0.005).

No serious muscarinic side effects such as bronchospasm and bradycardia were observed in this study. Typical muscarinic side effects (increased sweating, lacrimation, salivation, nausea, and diarrhea) were observed in all patients with blepharospasm within the first minute. These side effects did not always appear when the edrophonium injection was administered to patients with hemifacial spasms. The VAS scores of the subjective signs and the mVAS scores of the objective signs are presented in Figures 1, 2 respectively. Involuntary eyelid movements became obvious 4–8 min after edrophonium injection. In patients with blepharospasm, the VAS scores for the subjective signs and the mVAS scores for the objective signs were significantly higher after edrophonium injection than they were after saline injection (Figure 1). In patients with hemifacial spasms, no statistically significant difference was observed between the VAS and mVAS scores in the edrophonium injection and placebo groups (Figure 2). The relative values were significantly higher in patients with blepharospasm than they were in patients with hemifacial spasms (Figure 3).

## Discussion

We examined whether the signs of blepharospasm were amplified by edrophonium in this preliminary study. Both the subjective and objective signs of blepharospasm were amplified by edrophonium administration; in contrast, the signs of hemifacial spasm did not change after edrophonium administration. Moreover, typical muscarinic side effects were observed in all patients with blepharospasm. Collectively, these findings suggest that this challenge test may help to distinguish focal eyelid dystonia from other abnormal eye closing syndromes.

We also found that both the subjective and objective relative values were significantly higher in blepharospasm than they were in hemifacial spasm. Thus, comparing symptoms at the time of the saline injection may result in a more accurate diagnosis.

Edrophonium is a strong cholinergic drug; therefore, side effects such as bradycardia can occur following its administration. When conducting this study, we performed tests to exclude patients with heart disease, hence no serious muscarinic side effects were observed.

However, unlike the patients with hemifacial spasm, typical muscarinic side effects (increased sweating, lacrimation, and salivation) were observed in all patients with blepharospasm within the first minute. It has been reported that dystonia is caused by a mismatch between motor and sensory signals. The strong reaction against the edrophonium and the typical side effects that were observed following edrophonium administration may be signs of dystonia.

As mentioned previously, eyelid closure dysfunction is caused by abnormal functioning of the levator palpebrae superioris muscle or involuntary contraction of the orbicularis oculi muscle. In particular, abnormal functioning of the levator palpebrae superioris muscle is due to muscle disease, neuromuscular junction disease, or peripheral nerve disease. Our diagnoses are based on the results of blood tests, brain imaging studies, and electrophysiological tests. Since the edrophonium test has been established for diagnosing MG, we also examined patients with MG using the protocol of this study. However, we only examined three cases, because performing the edrophonium test in these patients takes longer than usual. We found that these three cases showed a tendency to improve following the edrophonium test (data not shown), similar to the results of previous studies (Juel and Massey, 2007).

On the other hand, involuntary contraction of the orbicularis oculi muscle occurs due to blepharospasm or hemifacial spasm. We did not observe changes in the symptoms of patients with hemifacial spasm. There is now considerable evidence that primary hemifacial spasms are, in almost all cases, related to the vascular compression of the facial nerve at its root exit zone from the brainstem (Abbruzzese et al., 2011). Cholinesterase inhibitors have been shown to improve the symptoms in patients with neuromuscular junction disorders, but they do not act on peripheral nerve diseases. Therefore, for patients with hemifacial spasms, it was predicted that the symptoms would not change, which is not contradictory to our findings. These results suggest that the edrophonium challenge test is helpful for distinguishing blepharospasm from bilateral hemifacial spasm.

The obvious symptoms induced by edrophonium are indirect proof that the anticholinergic drug is effective in patients who are edrophonium responders. It has been reported that eyelid closure improved following edrophonium injections that were administered for complications of MG and blepharospasm (Funakawa et al., 1992). In this case, the symptom improved by administering cholinergic drugs. Further research is necessary to establish whether the patients in whom the symptoms became obvious by edrophonium would respond to anticholinergic agents or not.

Multiple forms of dystonia are associated with genetic mutations, but the pathogenesis of sporadic dystonia is usually unclear. Abnormal plasticity in the basal ganglia (BG) is believed to be important to disease etiology, and acetylcholine is a critical neuromodulator in the BG (Peterson et al., 2010). There are two possible sites of action for edrophonium: the central nervous system (including the BG) or the peripheral nerves (Jankovic, 2009).

Edrophonium is hydrophilic and has limited capacity to cross the blood–brain barrier. In addition, edrophonium is rapidly excreted by the kidney, suggesting limited direct efficacy in the central nervous system (Back and Calvey, 1972; Calvey et al., 1976; Gemmill et al., 1988). It has been reported that edrophonium mitigates allodynia in an animal model of neuropathic pain, suggesting changes in afferent fiber transmission to the spinal cord that could in turn alter central sensorimotor integration (Hwang et al., 1999). The effects of edrophonium on the neuromuscular junction and autonomic ganglia have been studied, but those on the central nervous system have not been studied. In this study, dystonia became obvious 4–8 min after the edrophonium injection was administered, while the symptoms of MG improved within 1 min. This delay suggests that the effects of edrophonium on blepharospasm are centrally mediated by changes in afferent input.

In conclusion, we demonstrated that the edrophonium challenge test is helpful for distinguishing blepharospasm from hemifacial spasm. Using the edrophonium challenge test can help reduce misdiagnoses and improve treatment efficacy. However, because of the small sample size and the fact that we did not randomize the order of the saline and edrophonium treatments, independent replication of this study with a larger sample size and treatment randomization is warranted.

## Author contributions

SM, selection patients; NM, HK, and MT, video rater; YI, video editor; RK, general manager.

### Conflict of interest statement

The authors declare that the research was conducted in the absence of any commercial or financial relationships that could be construed as a potential conflict of interest. The reviewer XH and handling Editor declared their shared affiliation, although they are in different branches of the University and have no reporting relationship. The handling Editor states that the process nevertheless met the standards of a fair and objective review.

## Supplementary material

Supplementary Video 1 **Eyelid closure is evident in the video**.

Supplementary Video 2 **Eyelid closure is confirmed by edrophonium injection**.
